# The MD Anderson Algorithm for Lymphedema Management

**DOI:** 10.3390/jcm14061851

**Published:** 2025-03-10

**Authors:** Ashleigh M. Francis, Noa G. Kopplin, Edward I. Chang

**Affiliations:** Department of Plastic & Reconstructive Surgery, The University of Texas MD Anderson Cancer Center, Houston, TX 77030, USA; noa.g.kopplin@uth.tmc.edu (N.G.K.); eichang@mdanderson.org (E.I.C.)

**Keywords:** lymphedema, prevention, lymphovenous bypass, vascularized lymph node transfer

## Abstract

This article details the MD Anderson Cancer Center algorithm for lymphedema management. We discuss prophylaxis against and treatment options for both upper extremity and lower extremity lymphedema.

## 1. Introduction

Secondary lymphedema (LE) is a chronic, disabling condition that results from a disruption of the normal lymphatic system. LE often presents as swelling and fibrosis of the upper or lower extremity following surgery or radiation which impedes lymphatic drainage. Specifically, lymph node dissection or lymphadenectomy (LND) disrupts physiologic lymphatic flow and is a primary risk factor for LE. It is estimated that LE impacts around 2–3 million people in the US, with 200,000 new cases diagnosed annually [1,2]. LE most frequently results from cancer treatment in developed nations including the United States, with estimates that 15–20% of patients may develop LE [3,4]. Of the patients who develop LE, the majority will occur within two years of surgery; however, LE can develop many years after the initial cancer treatment [4,5]. Additionally, patients who undergo lymphadenectomy and radiation are at the highest risk for developing LE [6,7].

Developing LE has been shown to negatively impact patients both physically and psychologically, ultimately leading to reduced quality of life [8,9,10]. LE often leads to decreased mobility given additional limb weight and difficulty maneuvering with a swollen limb. Obesity is common in patients with LE, leading to further deleterious effects on mobility, body image, and quality of life. Once diagnosed with LE, conservative treatments may provide only a marginal benefit and largely aim at reducing symptoms and preventing further progression of edema. Conservative treatments are often time-consuming and focus on decongestive therapy such as compressive bandaging and garments or lymphatic massage to mitigate swelling. This highlights the growing need for interventions aimed at preventing the onset of lymphedema at the time of the initial surgery or to provide a physiologic means of improving the lymphatic drainage from the affected limb.

Recent advances in our understanding of the anatomy and physiology of the lymphatic system have yielded new surgical techniques that help to treat lymphedema and improve quality of life for patients. Several physiologic procedures have arisen as advantageous options for surgical treatment of lymphedema, including the lymphovenous bypass (LVB) and vascularized lymph node transfer (VLNT) approaches; these procedures serve to improve lymphatic fluid drainage in areas with restricted flow [3,11,12,13]. Vascularized lymph node flaps may be harvested from various donor sites, including the neck, groin, lateral thoracic region, and intraabdominally. Once the patient progresses to chronic lymphedema, which involves hypertrophy of fibroadipose soft tissues, suction-assisted lipectomy or excisional procedures are utilized to reduce the size and weight of the involved extremity. Despite the treatment modalities now readily available to surgeons, treatment can still be a challenging endeavor and prevention is preferred. The present summary aims to describe the MD Anderson algorithm for both surgical prevention and treatment of lymphedema in the upper and lower extremities.

## 2. Lymphedema Prevention

### 2.1. Upper Extremity

Upper extremity LE is a common complication of breast cancer treatment after axillary lymph node dissection (ALND), affecting roughly 21–25% of patients according to two recent meta-analyses [14,15]. Patients who undergo a combination of ALND with regional lymph node irradiation are at highest risk with up to 31.2% of patients developing LE at 5 years [14]. LE is not unique to breast cancer and can occur in other types of cancer as well, such as melanoma [3,16].

#### 2.1.1. Immediate Lymphatic Reconstruction (ILR)

Recent advances have focused on preventing LE by preserving lymphatic flow at the time when the regional lymphatics are disrupted during surgery. This was first described as the “Lymphatic Microsurgical Preventing Healing Approach” (LYMPHA) [17]. This technique aims to preserve lymphatic flow by taking a transected lymphatic vessel and surgically anastomosing it to a nearby vein, thereby preserving the lymphatic flow in that vessel. Further studies and meta-analyses have validated this protocol and have shown that immediate lymphatic reconstruction (ILR) can be effective in preventing both upper and lower LE when repaired at the time of lymphadenectomy [18]. However, one recent study failed to demonstrate any value and risk reduction in lymphedema for breast cancer patients undergoing ILR at the time of the axillary dissection [19].

**Figure 1:**
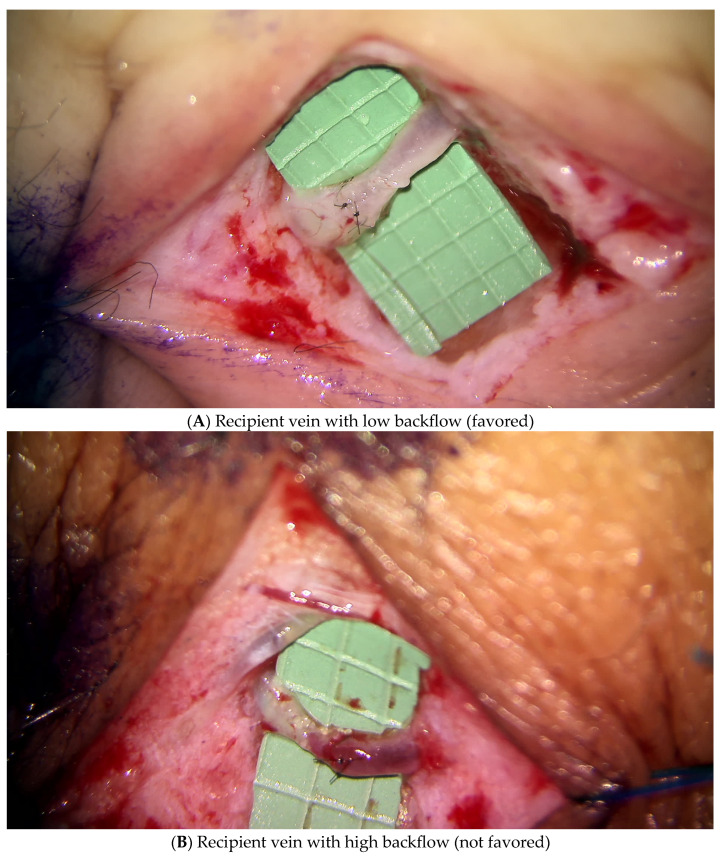
Recipient vein characteristics.

To perform ILR, a multidisciplinary approach has been adopted with the breast surgeons who perform axillary reverse mapping (ARM) during ALND to identify and preserve the lymphatics. Five to ten cc of isosulfan blue dye is injected into the dermis of the medial surface of the upper arm. As the dye disperses throughout the axillary lymphatic system, the surgical team is able to visualize and differentiate between the lymph nodes and vessels that drain the arm and breast [20]. The blue lymphatic vessels are clearly draining the upper extremity and are tagged for the ILR procedure. In addition to the identification of the lymphatic vessels, the breast surgeons are also cognizant to preserve branches of the axillary vein and thoracodorsal axis to serve as recipient veins. Once the ALND is complete, the microsurgeon will perform LVB which involves anastomosing a transected lymphatic vessel with a nearby recipient vein. The most commonly used recipient veins are branches off the axillary vein, which include the lateral thoracic vein, serratus branch of the thoracodorsal vein or one of the vena comitantes (VC) of the thoracodorsal artery. In general, at least one VC is preserved to preserve the option for harvesting a latissimus dorsi flap. In order to maintain physiologic lymphatic flow in an LVB, recipient veins with low backflow are chosen so that lymphatic fluid will drain from lymphatic vessel into the vein (Figure 1).

At our institution, we have implemented ILR in all breast cancer patients deemed high-risk for lymphedema, such as patients undergoing both ALND and radiation therapy or obese patients undergoing ALND. We have also begun employing this in truncal melanoma patients undergoing ALND, as these patients are also at high risk, given that level 3 lymph nodes are routinely removed. The resecting surgeons perform ARM to preserve the lymphatics, which are bypassed to a nearby vein. Typically, we perform bypasses for all lymphatics identified using 11-0 nylon in an end-to-end fashion. We generally prefer direct anastomosis of the lymphatics if the lymphatic is large enough and technically achievable. If the lymphatic is too small for direct anastomosis, the LYMPHA technique can be used, whereby the small caliber lymphatic vessel is intussuscepted into the recipient vein. In circumstances where a number of lymphatics are identified with few recipient veins, a multi-barrel anastomosis can also be performed. In general, the thoracodorsal pedicle is preserved to maintain the latissimus dorsi flap as an option for additional breast reconstruction. Postoperatively, patients are instructed to elevate the involved extremity as much as possible, and a range of motion protocol is implemented at 2 weeks. We perform volumetric measurements, perometry, and bioimpedance at 3 months, 6 months, 12 months, 18 months, and 24 months. ILR has reduced our lymphedema rates in this patient population from around 38% to 19% in our prospective study, which is ongoing.

#### 2.1.2. Vascularized Lymph Node Transfer (VLNT)

**Figure 2:**
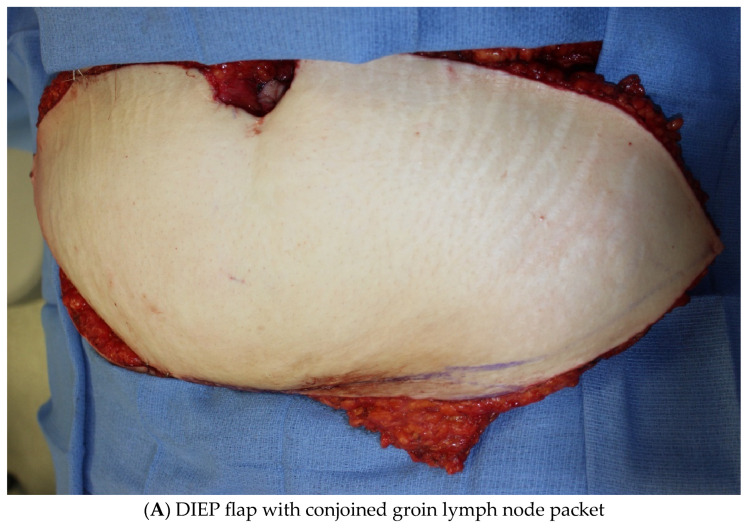

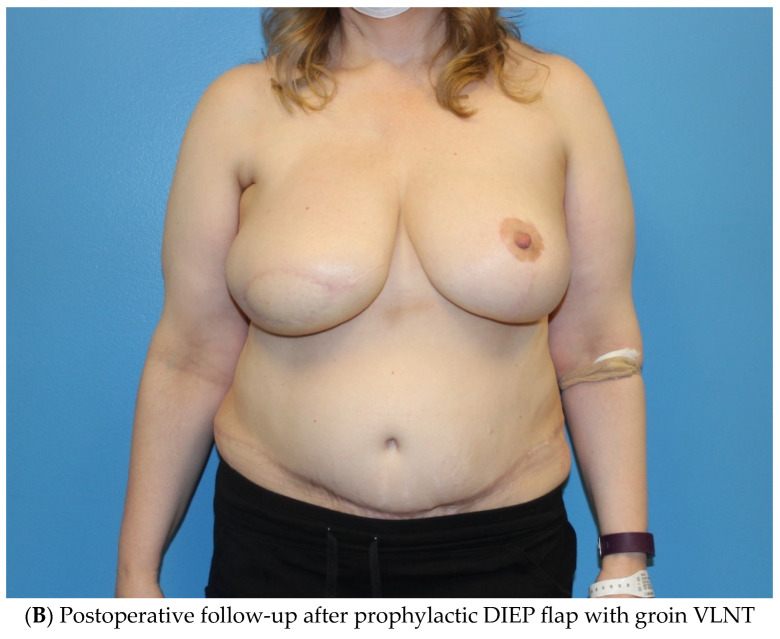
Prophylactic groin VLNT with DIEP flap.

While ILR with prophylactic LVB remains the workhorse of lymphedema prevention, VLNT remains a powerful tool for surgical treatment of LE to date. At MD Anderson, we have begun exploring the use of prophylactic groin VLNT combined with DIEP flap in patients desiring autologous breast reconstruction who carry a high risk of LE (Figure 2). We consider this only in high-risk breast cancer patients who have undergone both ALND and radiation who have the greatest chance of developing LE and are also seeking autologous breast reconstruction. The authors generally proceed with reconstruction six months after the completion of radiation therapy, which is still well within the window in which patients can develop LE. For patients who develop LE after undergoing autologous breast reconstruction with a DIEP flap, the groin lymph node flap is no longer a viable option for LE treatment, as its blood supply is divided during the DIEP flap, and alternate lymph node donor sites are offered. Several studies have established that combined groin VLNT with a DIEP flap is a valuable tool in therapeutic LE management [21,22,23]. Consequently, for high-risk patients seeking autologous breast reconstruction, we hypothesized that integrating prophylactic groin VLNT with a DIEP flap could potentially lower their risk of developing LE. The groin VLNT is harvested using reverse mapping and lymphoscintigraphy to ensure that the lymph nodes draining the lower extremity are not harvested, which could inadvertently cause LE of the lower extremity. To date, no cases of iatrogenic LE have occurred using these two modalities for groin VLNT harvest. The findings of our prospective study are forthcoming.

### 2.2. Lower Extremity

An inguinal lymph node dissection (ILND) is often indicated in patients diagnosed with metastatic penile, vulvar, anal, or skin cancers [24]. Unfortunately, there is a higher risk for developing LE after an ILND when compared to ALND, with incidence ranging from 18–64% [3,11,12,13]. In addition to LE, ILND is also associated with increased rates of wound complications such as infections, wound dehiscence, and seromas [11]. Recognizing the increased risk of LE and wound complications, we have begun to develop preventative treatment strategies to improve patient outcomes.

#### Immediate Lymphatic Reconstruction

**Figure 3:**
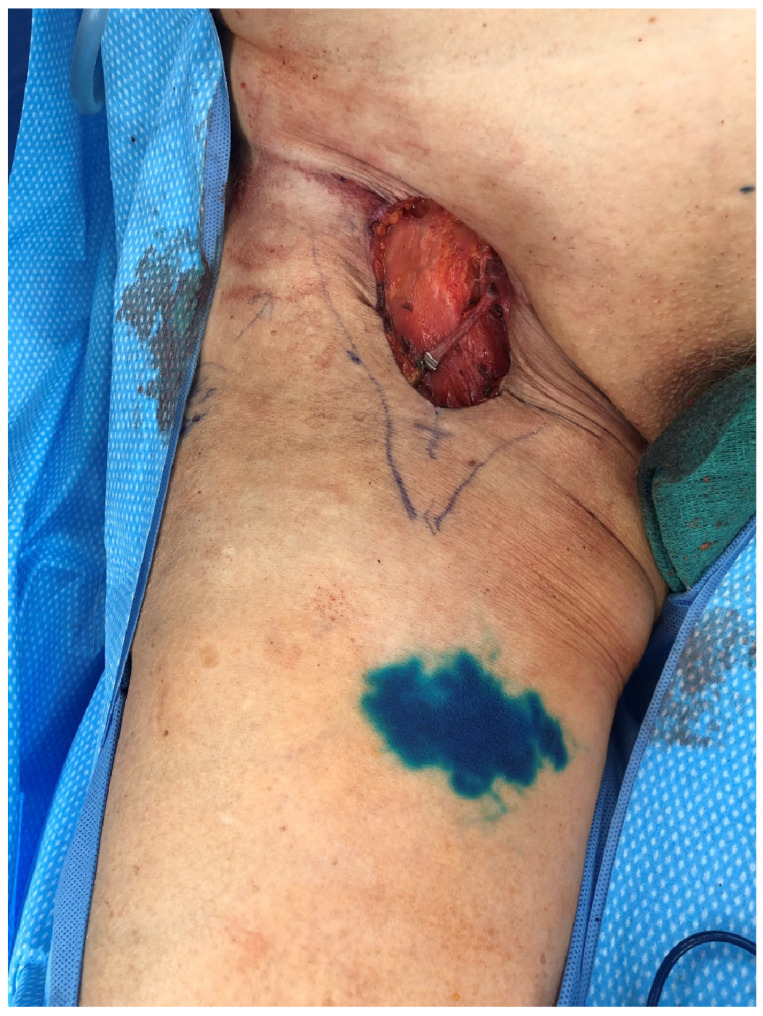
Inguinal reverse lymphatic mapping.

**Figure 4:**
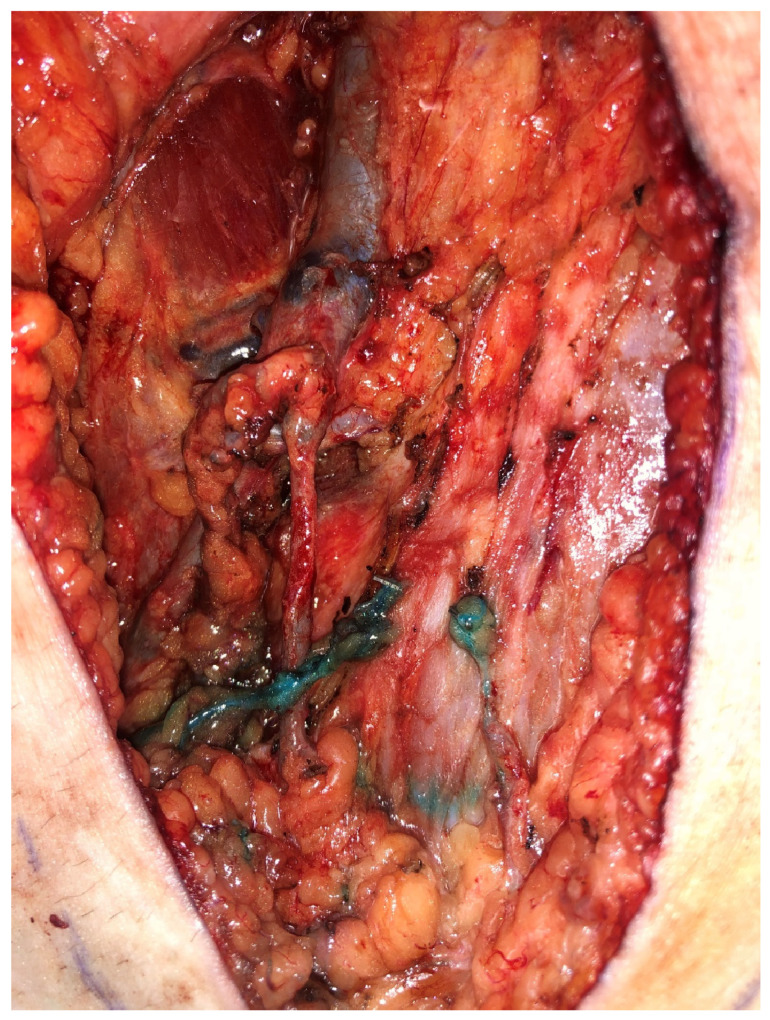
Identification of lymphatics (blue) and venous targets.

**Figure 5:**
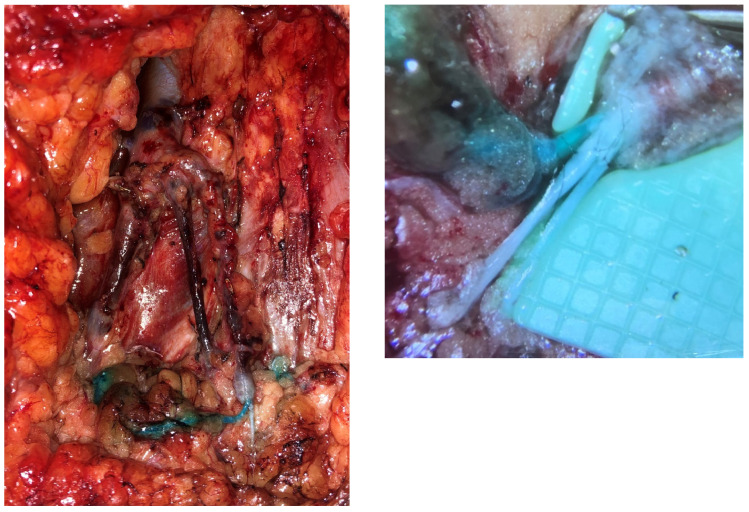
Prophylactic inguinal lymphovenous bypass—triple-barrel end-to-end anastomosis using SIEV as recipient vein.

Similar to the upper extremity, ILR has been shown to improve rates of LE when performed at the time of ILND, as documented by several studies [18,25,26,27]. ILR after ILND uses reverse inguinal lymphatic mapping (RIM) to identify lymphatics which are then bypassed to nearby recipient veins. RIM is performed in a similar fashion to ARM, with injection of the dye 10–12 cm inferior to the inguinal crease on the medial aspect of the thigh (Figure 3). In the groin, there are a multitude of recipient veins that can be utilized, including branches of the greater saphenous vein and external pudendal veins medially, and the superficial inferior epigastric vein (SIEV) and superficial circumflex iliac vein (SCIV) laterally (Figure 4). As with upper extremity ILR, the veins must be preserved with enough length to facilitate the LVB, so close collaboration and discussion with the resecting oncologic surgeon is crucial (Figure 5). Postoperatively, patients are instructed to elevate their leg as much as possible and initiate range of motion therapy at 2 weeks. A prospective trial is currently underway evaluating ILR in melanoma patients undergoing ILND, and data are forthcoming.

## 3. Lymphedema Treatment

### 3.1. Upper Extremity

A multidisciplinary approach is crucial to preventing and treating LE. Early physiotherapy greatly improves function, quality of life, mobility, and body image in breast cancer patients with axillary lymphadenectomy, as shown in a randomized clinical trial by Torres Lacomba et al., whereby LE rates decreased from 25% in the control group to 7% in the intervention group [28]. Similarly, physiotherapy continues to play a critical role in LE treatment once patients progress to a diagnosis of LE, whereby patients are optimized with complete decongestive therapy (CDT). Physiotherapists who perform CDT ensure patients are compliant with compression therapy, lymphatic massage, and pneumatic compression pumps. Once patients are optimized, they are referred for surgical treatment.

#### 3.1.1. Lymphatic Mapping and Lymphovenous Bypass (LVB)

Generally, indocyanine green (ICG) lymphatic mapping is performed in the operating room rather than in the clinic, as it can be quite painful for the patient. Patients who have subtle LE without extensive axillary deformity (hollowing or contracture) with less than 15% volume difference are candidates for LVB, alone or with VLNT. Patients with more advanced LE but who are opposed to a VLNT can opt to proceed with an LVB alone; however, in patients with advanced lymphedema, the likelihood of identifying a suitable lymphatic channel and significant improvement in these circumstances are marginal.

**Figure 6:**
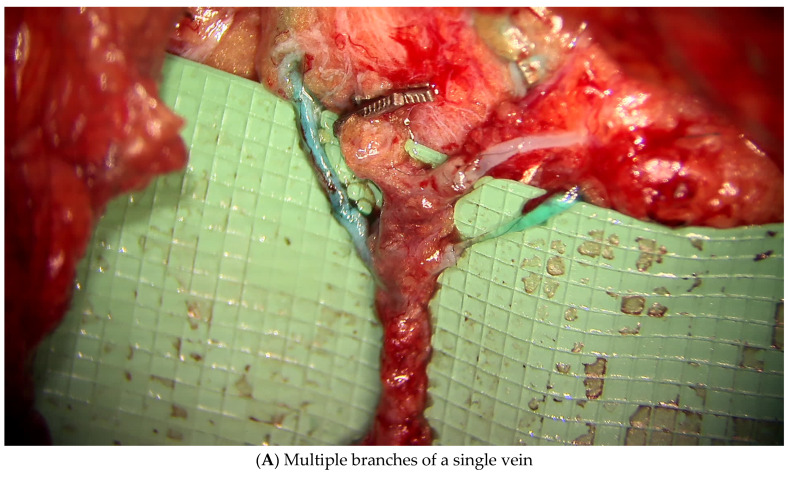

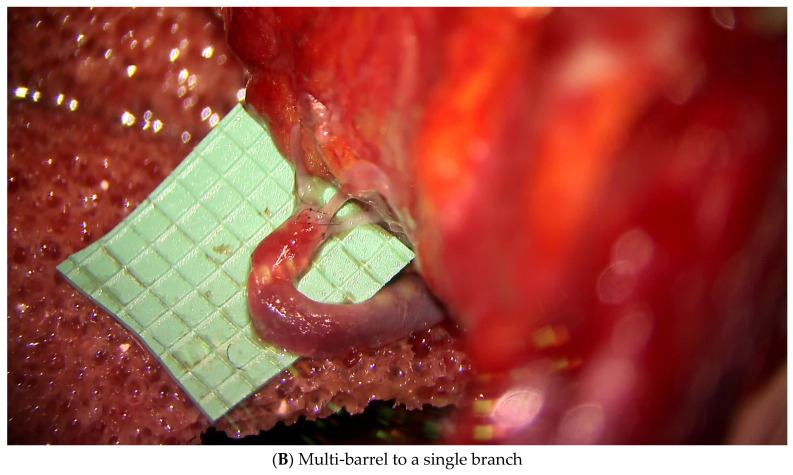
Different recipient end-to-end anastomotic configurations.

Patients are taken to the OR and placed under general endotracheal anesthesia. Then, 0.1 cc of ICG dye is injected into the finger web spaces and along the volar wrist crease at the radial and ulnar aspects to perform lymphatic mapping with the SPY system (Stryker, Kalamazoo MI). We grade all patients using the MD Anderson staging system. Lymphatic targets are identified and marked. A vein finder is used to identify nearby veins, and a local anesthetic containing epinephrine is injected along the incision for hemostatic purposes. A total of 0.1 cc of isosulfan blue dye is injected 2 cm distal to where the lymphatic has been identified. A 1 cm horizontal incision is made over the identified lymphatic and adjacent vein. A LVB is performed using a direct anastomosis with 11-0 nylon. While a number of different orientations can be used for the anastomosis, the end-to-end orientation has been found to have the highest long-term patency rates. Consequently, if only one nearby vein exists, then two options exist: (1) multiple anastomoses to different small branches off the vein; or (2) a multi-barreled approach, where multiple lymphatics are anastomosed in tandem to one vein (Figure 6). Once the anastomosis is completed, we confirm patency by visualizing blue dye or lymphatic fluid flowing into the vein. In general, functional lymphatics without evidence of obstruction are preserved (lymphatics with evidence of proximal flow on ICG mapping), and multi-level bypasses on the same lymphatic channel are not performed.

Postoperatively, the patients keep their upper extremity elevated as much as possible. We avoid any decongestive therapy measures until one month postoperatively, at which point they are allowed to resume all forms of therapy, which generally includes compression, lymphatic massage, and pneumatic compression pumps, without additional restrictions, until they no longer need it. Most patients are able to dramatically decrease their use of CDT, but they are still instructed to use compression when exercising or taking a flight.

#### 3.1.2. Vascularized Lymph Node Transfer (VLNT)

Like ILR, VLNT also aims at restoring physiologic lymphatic flow, which is disrupted by the removal of lymph nodes during oncologic resection or by radiation therapy, by transferring lymph nodes and restoring any volume deficits. VLNT was historically reserved for patients with more advanced stage lymphedema; however, our approach has evolved to combining both the LVB and VLNT for patients who opt to proceed with the more involved VLNT option. Furthermore, the need for volume replacement is often necessary following release of scar contractures following an axillary dissection, which has also demonstrated improvement in range of motion and lymphedema outcomes [29,30].

For women who suffer from breast cancer-related lymphedema (BCRL) and desire reconstruction, a DIEP flap with groin VLNT is our first choice. If the patient has undergone radiation, reconstruction with a VLNT is performed a minimum of 6 months after completion of radiation to minimize the risks of thrombotic complications and total flap loss [31]. The combined DIEP VLNT is also the recommended approach for women with LE who also suffer from complications or concerns with an implant-based reconstruction. To minimize the contour deformity in the groin donor site, a “flap” based on the upper abdominal skin incision can be designed to fill the donor site defect [32]. Alternatively, fat-grafting at the revision stage can also improve the groin donor site. Recently, we have again adopted a combined approach to treating patients with BCRL, where the DIEP VLNT approach is combined with an LVB as well. Studies have demonstrated that this combined technique is more effective than the DIEP VLNT alone [22,23].

For patients who do not need breast reconstruction, the omentum is our first choice for VLNT. In most cases, the omentum can be harvested with a minimally invasive approach, either laparoscopically or robotically. For women who have already had a DIEP flap, an open approach to harvest the omentum is performed through their previous DIEP scar. The omentum is our first-choice VLNT donor site as it is a lymphatic-rich organ with a dual-blood supply which allows for a dual-level lymph node transfer. This is particularly advantageous in women with lymphedema distally at the level of the forearm or hand. The omentum can be split into two independent VLNT and placed into the axilla and the distal forearm. The larger portion of the omentum is typically anastomosed to the thoracodorsal vessels and placed in the axilla. In women with a partial mastectomy defect or deformity following previous breast reconstruction, the omentum can also be used to restore volume and improve the lymphedema. The flap is buried and monitored with an implantable doppler. When counseling patients, they should be informed that a dual-level transfer does require a scar on the forearm and sometimes a small skin graft, which some women dislike from an aesthetic perspective. The main concern of the omental flap is that it requires an intra-abdominal harvest, which may carry risks of injury to other structures, an incisional hernia, or the need to convert from a minimally invasive approach to an open harvest.

For patients who are not candidates for an omental harvest (extensive previous abdominal surgery or known omentectomy), other VLNT donor sites include the submental, supraclavicular, groin, or lateral thoracic lymph node basins. These tend to be smaller lymph node flaps are less suitable for volume replacement as compared to the omentum. Each lymph node flap donor site has its advantages and disadvantages so the patient should always be involved in the decision-making process of which VLNT flap they most favor as the efficacy is relatively equivalent for any donor site in terms of improving patients’ lymphedema [33]. The lateral thoracic lymph node flap is our last-choice flap as it carries a high-risk of donor site lymphedema and has been shown to be the least effective donor site option [34].

When performing a VLNT, we always approach the axilla thoughtfully and carefully. A full scar release is preferable if it can be achieved safely, as this generally allows for better lymphatic drainage and helps to improve the range of motion. The thoracodorsal vessels are our first choice of recipient vessels and should be traced as far proximally as can be achieved without injuring the axillary vein. For the omental flap, we prefer a second venous outflow, when possible, as these flaps have a higher risk of venous congestion given their dual blood supply and thin-walled, highly distensible veins.

Postoperatively we keep the patients hospitalized for 4 to 5 days with their upper extremity elevated and abducted from their body. They are instructed to limit arm elevation to the level of the shoulders and are also advised to avoid any decongestive therapy measures until one month postoperatively, at which point they are allowed to resume all forms of therapy including compression, lymphatic massage, and pneumatic compression pumps. Patients are followed postoperatively according to our previously described protocol.

### 3.2. Lower Extremity

#### 3.2.1. Lymphatic Mapping and Lymphovenous Bypass (LVB)

For patients with lower extremity LE, patients again can opt for a LVB or a combined LVB/VLNT approach. Most patients with lower extremity LE will experience some improvement with LVB; however, lower extremity disease presents more challenges as elevation of the lower extremity is more difficult to maintain than the upper extremity. The lymphatic mapping is again performed in the operating room, similarly to that performed for patients with upper extremity LE, with the injections performed in the webspaces of the feet and at the ankle crease. The bypasses are performed again in a similar fashion, from a technical standpoint, to how they are for the upper extremity.

#### 3.2.2. Vascularized Lymph Node Transfer (VLNT)

Patients are given 6 months to determine whether they have improved after bypasses. If they fail to improve or fail to improve to the degree desired, then we proceed with VLNT. While any VLNT donor site can be used, we typically do not offer an inguinal lymph node transfer, and the omentum is our first choice. Even more so than the upper extremity, the omentum is an excellent choice because the omentum again can be split into two independent flaps, so patients may receive a dual-level transfer given the dependent nature of the lower extremity. For the proximal anastomosis, we generally prefer the descending branch of the lateral circumflex femoral artery (LCFA) and vein for the recipient vessels. If a flow-through orientation can be performed for the artery, we will typically use the distal continuation of the LCFA or branches off the femoral artery if the flap is tunneled proximally into the groin. Some subcutaneous tissue is excised in the medial distal leg to create space for a dual-level transfer. The smaller of the two omental flaps is placed in the distal leg, typically using the posterior tibial vessels, either end-to-side or as a flow-through flap. The flap is usually buried but a skin graft may be occasionally necessary, similarly to distal placements in the upper extremity. As with any free tissue transfer, monitoring is critical and an implantable doppler is routinely placed for buried omental flaps.

Postoperatively, we keep the patients hospitalized on bedrest for five days with their lower extremity elevated. At five days, a dangling protocol is initiated, where patients are gradually allowed to increase the duration of placing the leg in a dependent position, as well as a gradual increase in their weight-bearing status. Again, as with the upper extremity, patients are instructed to avoid any compression for four weeks, after which time they are allowed to resume compression, wrapping, and pumping as necessary. Again, SAL is an adjunct to a physiologic procedure and performed in a staged fashion. All patients are kept on oral antibiotics for one week following any invasive procedure performed on the lymphedematous limb.

### 3.3. Adjunctive Procedures

#### 3.3.1. Suction-Assisted Lipectomy

At MD Anderson, suction-assisted lipectomy (SAL) is typically only used as the primary treatment modality when patients are not candidates for a physiologic approach, although one study has shown SAL to have a strong effect on long-term volume reduction, even without any physiologic procedures [35]. We prefer to proceed with physiologic procedures such as LVB and VLNT as first line therapy, and secondarily, SAL is offered as an adjunct to a physiologic operation in a staged fashion. We rarely perform SAL at the same time as an LVB or VLNT. Typically, the second stage debulking operation is performed at earliest 6–12 months following their last physiologic procedure after confirming their measurements have plateaued before offering this as an option. In the operating room, we use epinephrine containing tumescent solution rather than tourniquet to complete SAL. We also use lymphatic mapping to confirm that we are not performing SAL in an area where good lymphatic drainage is visualized or near a previous LVB. Interestingly, however, the fear of worsening lymphatic drainage with SAL by damaging functioning lymphatics has been called into question and may be more of a theoretical risk than an actual risk [36].

#### 3.3.2. Excisional Procedures

Typically, we reserve excisional procedures as a primary treatment for lymphedema only for patients who are not candidates for a physiologic approach or SAL. These tend to be patients with very advanced lymphedema with extensive fibrosis, where excisional debulking is the only option. The Charles procedure, while now less commonly used in the current era given the advent of LVB and VLNT, remains an important option in very severe cases of LE [37]. Additionally, some authors support the combined use of physiologic and excisional procedures as a mainstay for therapy, although we have not yet employed that option at our institution [38].

## 4. Conclusions

Secondary lymphedema affects a large proportion of cancer patients and has a significant detrimental impact on quality of life, discomfort, body image, function, and mobility for these patients. In recent years, the number of surgical options for treatment of lymphedema has blossomed, allowing us to make great strides in how we manage these patients and what types of procedures we are able to offer them to help them regain some semblance of their lives and not be consumed by the deleterious effects of LE. Although there are various surgical methods offered in the current era, their efficacy is still being tested and more studies with standardized protocols are needed to define the true medium- and long-term effects of these interventions. Furthermore, we need to continue to perfect the multidisciplinary surgeon-physiotherapist protocol for the management and prevention of lymphedema. As the field grows and more advancements and novel approaches emerge, our algorithm continues to evolve, enabling us to provide the best care possible for this challenging patient population and optimize their outcomes.

## Data Availability

No specific data were mentioned in this manuscript as it is a review of our current algorithm for treatment of lymphedema.
